# Mosquito host choices on livestock amplifiers of Rift Valley fever virus in Kenya

**DOI:** 10.1186/s13071-016-1473-x

**Published:** 2016-03-31

**Authors:** David P. Tchouassi, Robinson O. K. Okiro, Rosemary Sang, Lee W. Cohnstaedt, David Scott McVey, Baldwyn Torto

**Affiliations:** International Centre of Insect Physiology and Ecology, P. O. Box 30772-00100, Nairobi, Kenya; Centre for Virus Research, Kenya Medical Research Institute (KEMRI), P.O. Box 54840-00200, Nairobi, Kenya; United States Department for Agriculture - Agricultural Research Station (USDA-ARS), Arthropod-Borne Animal Disease Research Unit, Center for Grain and Animal Health Research, 515 College Ave, Manhattan, KS 66502 KS USA

**Keywords:** Attractancy, Engorgement rate, RVF livestock amplifiers, Enclosure trap, Surveillance

## Abstract

**Background:**

Animal hosts may vary in their attraction and acceptability as components of the host location process for assessing preference, and biting rates of vectors and risk of exposure to pathogens. However, these parameters remain poorly understood for mosquito vectors of the Rift Valley fever (RVF), an arboviral disease, and for a community of mosquitoes.

**Methods:**

Using three known livestock amplifiers of RVF virus including sheep, goat and cattle as bait in enclosure traps, we investigated the host-feeding patterns for a community of mosquitoes in Naivasha, an endemic area of Rift Valley fever (RVF), in a longitudinal study for six months (June–November 2015). We estimated the incidence rate ratios (IRR) where mosquitoes chose cow over the other livestock hosts by comparing their attraction (total number collected) and engorgement rate (proportion freshly blood-fed) on these hosts.

**Results:**

Overall, significant differences were observed in host preference parameters for attraction (F_2,15_ = 4.1314, *P* = 0.037) and engorgement (F_2,15_ = 6.24, *P* = 0.01) with cow consistently attracting about 3-fold as many mosquitoes as those engorged on sheep (attraction: IRR = 2.9, 95 % CI 1.24–7.96; engorgement: IRR = 3.2, 95 % CI = 1.38–7.38) or goat (attraction: IRR = 2.7, 95 % CI 1.18–7.16; engorgement: IRR = 3.28, 95 % CI 1.47–7.53). However, there was no difference between the attraction elicited by sheep and goat (IRR = 1.08; 95 % CI 0.35–3.33 or engorgement rate (IRR = 0.96, 95 % CI  0.36–2.57).

**Conclusion:**

Despite the overall attractive pattern to feed preferentially on cows, the engorgement rate was clearly independent of the number attracted for certain mosquito species, notably among the flood water *Aedes* spp., largely incriminated previously as primary vectors of RVF. Our findings suggest that insecticide treated cattle (ITC) can be exploited in enclosure traps as contact bait in the monitoring and control of disease-causing mosquitoes in RVF endemic areas.

## Background

The foraging behaviour of disease vectors controls the opportunities for infection and transmission of pathogens that cause vector-borne diseases [1]. The emergence of zoonotic arbovirus diseases is intimately linked to the range of blood hosts that may be fed upon by vectors such as mosquitoes [2]. As such, assessment of host blood feeding preference is of ecologic and epidemiological value for arboviral zoonoses such as Rift Valley fever (RVF), a disease of public and veterinary health importance transmitted by mosquitoes. Despite the isolation of the causative agent, RVF virus, from so many mosquito species [3–5], knowledge of their blood- feeding patterns on potential vertebrate hosts remains poor and is only just beginning to be appreciated.

A number of methods have been employed to examine the host preferences of RVF mosquitoes. Earlier studies employed biochemical and molecular identification of host source from blood in naturally engorged mosquitoes sampled using traps [6, 7]. Evaluation of host preference based on trap captures of mosquito vectors is limited because certain important species may not be readily collected in traps. For example, during the RVF outbreak of 2006/07, there was the lack of blood-fed *Culex* spp. mosquitoes sampled, yet these species constituted a large proportion of mosquitoes sampled during the period [5]. While this points to the inefficiency of traps to representatively sample different species, this observation inadvertently fails to add much to our knowledge of the host feeding patterns of *Culex* spp., which have been incriminated as secondary vectors of the disease. Additionally, earlier studies on host preference have narrowly focused on selected flood water *Aedes* spp. mosquitoes incriminated as primary RVF vectors e.g. *Aedes mcintoshi* and *Aedes ochraceus*, in a specific geographic area. However, similar data remains wanting for other important species given that mosquito species incriminated in virus transmission vary from region to region [5] and not necessarily the floodwater *Aedes* spp. A number of samples processed for blood meal analyses may largely remain unidentified using biochemical and molecular means likely to be affected by integrity of the sample, quality and quantity of the DNA following blood meal digestion and specificity of the associated gene target [8].

Behavioural observations using choice assays in the field may provide a more objective tool for assessing host preference [9]. These competitive experiments may often represent what a mosquito experiences in nature when a host-seeking mosquito encounters more than one potential host source. Within this context, animal-baited trapping systems may be applicable to examine host associations of animal-biting insects and to determine the seasonal activity or geographic distribution of these insect species [10]. Animal-baited traps provide olfactory cues from hosts for attraction of mosquitoes [10, 11] and often overcome inherent biases from conventional mosquito traps and attractants. Using such methods the possibility of collecting host specific mosquitoes not readily encountered in conventional traps remains high. For instance, collections of mainly ornithophilic species of *Culex* or *Culiseta* has been achieved by baiting traps with birds [12, 13], which are not readily collected in conventional traps [14]. For vector species, animal-baited trapping is also useful for measuring parameters of pathogen transmission, including host feeding preference and host biting rate [10].

In a related study, animal skin host cues have been used as bait in conventional traps to evaluate host preference of RVF mosquitoes in the field [11, 15]. However, this approach is only suitable for host-attraction studies and provides no measure of engorgement. In studies of host preference the final criterion of host selection is taking a blood meal and the most epidemiological significant end point [16, 17]. Animal species may vary in attraction and acceptability and the knowledge is important in determining vector biting rates and exposure to pathogens and for risk assessment among farm animals. These parameters were also not monitored in a study using humans and calves as bait to evaluate the biting habits of mosquitoes associated with flooded dambos with particular interest in flood water *Aedes*, incriminated as primary RVF vectors [18]. Although livestock hosts (cow, sheep and goat) serve as amplifiers for RVF virus [19], we posit that the feeding parameters, attraction and engorgement, vary for a community of mosquitoes that could likely predispose them to differential risk of or source of infection. Hence, their assessment may help identify the biting pressure and the mosquito species feeding on these domestic animals and identify those most likely to transmit pathogens. Of epidemiological value, the highly attractive and acceptable host is the individual with the greatest potential exposure to risk of infection with RVF and possibly other mosquito-borne disease agents. Such an attractive host also serving as amplifier could facilitate enhanced transmission through infection of efficient and inefficient mosquito vectors. This knowledge can potentially be exploited in mitigation strategy against this disease to target the vectors given that effective vaccine or therapeutic treatments are lacking.

## Methods

### Study site

We carried out this study in the outskirts of Naivasha, an endemic site for RVF [20] where the first case of RVF was reported in Kenya. Naivasha is located in Nakuru County at an altitude of 1,884 m above sea level with an estimated 181,966 inhabitants as per the 2009 census. The climate is warm and temperate with an average annual temperature of 17 °C and rainfall of about 1, 150 mm with bimodal peaks experienced every March-May and October-December. The vegetation is characterized by patterns of shrub savannah, shrub and bush land and irrigated cropland. Among the inhabitants are the indigenous Masai who are predominantly pastoralists while the immigrants practice rain-fed and irrigated farming that includes the large multinational owned flower and horticulture farms nestled along the shores of Lake Naivasha, taking advantage of the fertile volcanic soils. The horticulture farming forms the main agricultural activity in the area, which is also rich in wildlife. Field experiments with animals were conducted in a farm in Maai Mahiu village located at 01°02.808’S, 036° 35.177’E.

### Study design

**Fig. 1:**
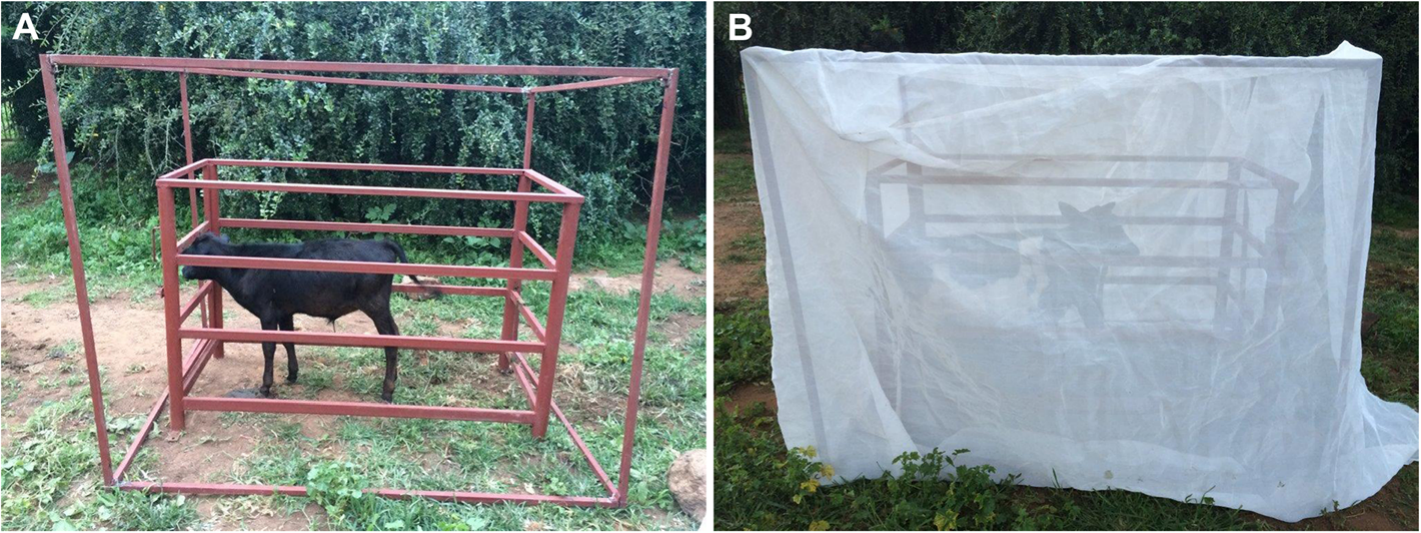
Experimental set up showing (**a**) animal (cow) restrained inside a cage and (**b**) enclosure trap with an opening for access of host-seeking mosquitoes to the animal

We used cow (*Bos taurus*), sheep (*Ovis aries*) and goat (*Capra hircus*) as bait in an enclosure trap comprising a cage measuring 1.83 m long × 1.83 m wide × 1.68 m high (cow) and 1.52 m long × 1.50 m wide × 1.78 m high (goat and sheep) and covered with fine and hard mesh-like netting material (Fig. 1). During each experiment, the enclosure trap was placed over each bait animal, which was restrained at the centre in an iron cage that allowed the animal space to freely move around (Fig. 1). Animals were placed in their respective cages at 19:00 h; thereafter, a side slot was opened, allowing host-seeking mosquitoes access to the animal baits.

Mosquitoes were then collected with a battery-powered backpack aspirator with the aid of a flashlight from the interior wall of the enclosure netting. Mosquitoes were arbitrarily collected at 22:00 and 05:00 h the following morning although the samples were pooled to constitute each day’s collection based on total numbers aspirated at these times. Daily experiments comprised of using each animal as bait replicated over 8 days and typically conducted in the last week of every month from June to November 2015. During each experimental night, the animal-baited traps were placed at least 50 m apart employing a Latin square design in the trap placement in a uniform area in terms of vegetation cover. These animals were randomly selected from a herd of the same species usually held in pens throughout the night in the farm after grazing during the day accompanied by 1 or 2 herdsmen after which they returned to their homes 2–3 km away. The animals used were about a year old for goat and sheep just as the case of the calf (cow), which was easy to handle. Experiments were conducted 1 km from the pens where they are normally held at night.

### Mosquito processing and data analyses

Trapped mosquitoes were knocked down using triethylamine (TEA) and stored in liquid nitrogen. Once transported to the laboratory at the International Centre of Insect Physiology and Ecology (*icipe*), the samples were stored at −80 °C until identification. The species and the numbers of mosquitoes collected in each animal-baited trap were identified to species level using morphological keys of Edwards [21], Gillies & de Meillon [22] and Jupp [23]. The total number of mosquitoes collected per animal was defined as the number of mosquitoes ‘attracted’ to that host. The number found to be fully freshly blood fed were scored as ‘engorged’ and expressed as a proportion of the mosquito captures and for each species from each host species. Engorgement was determined by the distended abdomens and appearance of visible red blood coloration of engorged mosquitoes [24]. Partially engorged individuals were also classified as engorged. Data were analysed using R version 3.2.3 [25] at α = 0.05 level of significance. Data on the overall mosquito counts were compared using Chi - square test and a Generalized Linear Model (GLM) by fitting a quasipoisson for all mosquito species combined and separately for select species, with mosquito abundance as the only factor in the model after controlling for sampling period. We computed the diversity of community of mosquitoes by estimating and comparing the Shannon's diversity index using ANOVA by specifying the sampling period and total counts as explanatory variables. Overall number of engorged mosquitoes (and for species fairly represented across the sampling periods) out of the total sampled for each month was analysed using a Generalized Linear Model (GLM) with quasibinomial error and log link. Incidence rate ratios (IRR) and corresponding confidence interval (CI) were estimated against a reference category from a select animal type. Chi - square goodness-of-fit was used to compare the proportion engorged among the animal types for species not represented each sampling month.

### Ethical considerations

The study was approved by the Kenya Medical Research Institute Animal Use and Care committee (KEMRI-ACUC) and the Ethical Review Committee. Informed consent was obtained from the owner of the farm after explaining the background and objectives of the study.

## Results

A total number of 2,514 mosquitoes belonging to twenty-seven species in five genera were collected. Of these, 1,471 were found to be engorged with an overall engorgement rate of 58.5 %. The total number collected and number engorged for each species is presented in Table 1. *Aedes dentatus*, *Culex pipiens*, *Culex vansomereni*, *Anopheles gambiae* and *Anopheles funestus* were the most abundant species in their respective genera.

**Table 1:** Species composition and number attracted (number engorged) per host treatment from enclosure traps in Naivasha, Kenya

Species groups	Species	Cow	Sheep	Goat	No. collected(No. engorged)	Percentage engorged
Flood water *Aedes* spp.	*Aedes dentatus*	125 (92)	51 (24)	130 (57)	306 (173)	56.5
	*Aedes mcintoshi*	5 (4)	4 (3)	4 (3)	13 (10)	76.9
	*Aedes tarsalis*	27 (22)	8 (7)	13 (11)	48 (40)	83.3
	*Aedes tricholabis*	38 (19)	23 (3)	20 (2)	81 (24)	29.6
	*Aedes hirsutus*	3 (1)	0 (0)	4 (2)	7 (3)	42.9
*Culex* spp.	*Culex pipiens (s.l.)*	166 (76)	107 (37)	62 (11)	335 (124)	37.0
	*Culex vansomereni*	244 (90)	51 (8)	52 (12)	347 (110)	31.7
	*Culex univittatus*	17 (7)	12 (1)	7 (0)	36 (8)	22.2
	*Culex zombaensis*	186 (127)	36 (14)	45 (9)	267 (150)	56.2
	*Culex terzii*	88 (84)	84 (42)	55 (30)	227 (156)	68.7
	*Culex theileri*	39 (32)	1 (0)	8 (6)	48 (38)	79.2
	*Culex poicilipes*	6 (3)	0 (0)	0 (0)	6 (3)	50.0
	*Culex annulioris*	0 (0)	1 (0)	1 (0)	2 (0)	0.0
	*Culex rubinotus*	2 (1)	0 (0)	0 (0)	2 (1)	50.0
	*Culex ethiopicus*	2 (1)	0 (0)	1 (0)	3 (1)	33.3
	*Culex tigripes*	7 (0)	8 (1)	9 (0)	24 (1)	4.2
Anophelines	*Anopheles gambiae (s.l.)*	180 (161)	26 (12)	38 (18)	244 (191)	78.3
	*Anopheles funestus*	292 (277)	72 (50)	64 (45)	428 (372)	86.9
	*Anopheles coustani*	26 (21)	9 (6)	12 (9)	47 (36)	76.6
	*Anopheles maculipalpis*	1 (1)	2 (2)	6 (4)	9 (7)	77.8
	*Anopheles christyi*	6 (6)	1 (1)	0 (0)	7 (7)	100.0
Others	*Aedes (Stegomyia) chaussieri*	8 (3)	0 (0)	3 (2)	11(5)	45.5
	*Aedes (Stegomyia) aegypti*	2 (2)	1 (0)	5 (3)	8 (5)	62.5
	*Aedes (Stegomyia)* sp.	0 (0)	0 (0)	1 (1)	1 (1)	100.0
	*Aedes (Diceromyia) furcifer*	2 (2)	1 (1)	0 (0)	3 (3)	100.0
	*Aedeomyia furfuria*	0 (0)	3 (1)	0 (0)	3 (1)	33.3
	*Aedeomyia africana*	0 (0)	1 (1)	0 (0)	1 (1)	100.0

The number of mosquitoes attracted and the percentage engorged from each host showed that overall, most species preferred cattle over sheep or goat (Table 1). While the data suggest less preference for the small ruminants (goat and sheep), these end points varied depending on the species. Overall, attraction was highest for cow (1,472) followed by goat (540) and sheep (502), which was also reflected in the engorgement rate with cow recording the highest (70.1 %, *n* = 1,032) followed by sheep (42.6 %, *n* = 214) and goat (41.7 %, *n* = 225).

Overall, attraction significantly varied among the animals (*F*_2,15_ = 4.13, *P* = 0.037) and for all monthly trapping periods. The cow consistently attracted about 3-fold more mosquitoes than sheep (IRR = 2.9; 95 % CI 1.24–7.96) or goat (IRR = 2.7; 95 % CI 1.18–7.16). Data summary for the total number of mosquitoes attracted and percentage engorged for each sampling period and animal used are presented in Table 2. After controlling for the sampling period, a binomial regression model revealed that the engorgement rate significantly varied among the animals (*F*_2,15_ = 6.24, *P* = 0.01) and similarly, this parameter was about 3-fold higher for cow than sheep (IRR = 3.2, 95 % CI  1.38–7.38) or goat (IRR = 3.28; 95 % CI 1.47–7.53). However, there was no difference between sheep and goat in attraction (IRR = 1.08; 95 % CI 0.35–3.33) or engorgement rate (IRR = 0.96; 95 % CI  0.36–2.57).

**Table 2:** Seasonal variation in mosquito abundance and number engorged (%) per host treatment collected from enclosure traps in Naivasha, Kenya

Sampling period	Cow		Sheep		Goat	
	No. collected	No. engorged (%)	No. collected	No. engorged (%)	No. collected	No. engorged (%)
June 2015	208	155 (74.5)	100	42 (42.0)	184	76 (41.3
July 2015	212	166 (78.3)	47	18 (38.3)	50	10 (20.0)
August 2015	130	86 (66.2)	54	22 (40.7)	36	19 (52.8)
September 2015	93	80 (86.0)	27	16 (59.3)	46	35 (76.1)
October 2015	265	243 (91.7)	45	33 (73.3)	38	28 (73.7)
November 2015	564	302 (53.5)	229	83 (36.2)	186	57 (30.6)
Overall total	1472	1032 (70.1)	502	214 (42.6)	540	225 (41.7)

In studies of host preference the final criterion of host selection is taking a blood meal [16]. Because of significant difference in mosquito diversity estimated based on the Shannon's diversity index (*F*_5_ = 3.113; *P* = 0.0497), we compared the proportion of engorged mosquitoes (of the total collected) for selected species which were fairly represented across all the months viz: *Cx. pipiens*, *An. gambiae*, *An. funestus*, and *Ae. dentatus* (Table 3). Our analyses showed significantly higher numbers of *An. gambiae* feeding on cow than any of the small ruminants with about a 10- and 9-fold increase relative to sheep (IRR = 9.88; 95 % CI  3.33–30.50) or goat (IRR = 9.4; 95 % CI 3.65–25.24), respectively. There was, however, no difference in the proportion engorged between goat and sheep (IRR = 1.05; 95 % CI  0.31–3.57). An analogous pattern was observed for *An. funestus* as this species was 8-times more likely to feed on cow relative to sheep (IRR = 8.13;95 % CI  3.13–22.12) or goat (IRR = 7.80; 95 % CI 2.89–21.81) with no apparent difference in feeding rates between sheep and goat (IRR = 1.04;95 % CI  0.39–2.82). No significant difference among the animals in the proportion of engorged *Ae. dentatus* (*F*_2,14_ = 3.15; *P* = 0.07) and *Cx. pipiens* (*F*_2,14_ = 2.13, *P* = 0.16) (Table 4).

**Table 3:** Seasonal variation in abundance (number engorged) and comparison in engorgement rate across the host types for select species fairly represented throughout the sampling period

Species	Animal	June 2015	July 2015	August 2015	September 2015	October 2015	November 2015	*F*-test	*P*-value
*Culex pipiens*	cow^a^	39 (53)	7 (26)	15 (35)	6 (14)	4 (7)	5 (31)	F_2,14_ = 2.13	*P* = 0.16
	sheep^a^	7 (24)	2 (16)	7 (25)	2 (7)	2 (7)	17 (28)		
	goat^a^	6 (16)	0 (22)	3 (10)	1 (6)	0 (0)	1 (8)		
*Aedes dentatus*	cow^a^	42 (62)	12 (12)	11 (13)	8 (9)	18 (22)	1 (7)	F_2,14_ = 3.15	*P* = 0.07
	sheep^a^	14 (21)	1 (1)	3 (3)	0 (0)	3 (3)	3 (23)		
	goat^a^	42 (99)	2 (2)	2 (4)	4 (4)	3 (3)	4 (18)		
*Anopheles gambiae*	cow^a^	25 (26)	104 (114)	12 (14)	3 (3)	3 (3)	14 (20)	F_2,12_ = 15.59	*P* = 0.0005
	sheep^b^	4 (12)	7 (11)	1 (2)	0 (0)	0 (0)	0 (1)		
	goat^b^	7 (15)	6 (16)	1 (1)	2 (2)	0 (0)	2 (4)		
*Anopheles funestus*	cow^a^	23 (24)	23 (25)	32 (35)	46 (48)	153 (160)	0 (0)	F_2,11_ = 19.94	*P* = 0.001
	sheep^b^	10 (20)	2 (4)	9 (15)	7 (8)	22 (25)	0 (0)		
	goat^b^	10 (17)	0 (0)	3 (8)	12 (14)	20 (25)	0 (0)		

Host followed by the same letters indicate no significant difference in the proportion of engorged for each type following chi square goodness-of-fit at α = 0.05 level

**Table 4:** Comparison in the proportion engorged among the host treatments for select RVFv species in low occurrence

	Cow	Sheep	Goat		
Species	No. engorged (No. collected)	No. engorged (No. collected)	No. engorged (No. collected)	*χ* ^2^, df = 2	*P*-value
*Culex vansomereni*	90 (244)	8 (51)	12 (52)	10.856	0.004
*Culex zombaensis*	127 (186)	14 (36)	9 (45)	39.361	<0.001
*Culex theileri*	32 (39)	0 (1)	6 (8)	4.081	0.13
*Culex terzii*	84 (88)	42 (84)	30 (55)	48.097	<0.001
*Anopheles coustani*	21 (26)	6 (9)	9 (12)	0.76462	0.6823
*Aedes mcintoshi*	4 (5)	3 (4)	3 (4)	0.043333	0.9786
*Aedes tarsalis*	22 (27)	7 (8)	11 (13)	0.18205	0.913

For less abundant species with a history of RVF virus based on isolations (Table 4) (EFSA [3]) except for *Cx. terzii* (without prior association with RVF virus), a significant proportion were more likely to feed on cows than the other hosts. This was the case for *Cx. zombaensis*, *Cx. vansomereni* but not for *Ae. tarsalis*, *Ae. mcintoshi*, *An. coustani*, *Cx. theileri* and *Cx. terzii.*

## Discussion

Many of the species trapped are of particular significance as they have been incriminated as RVF vectors mainly on the basis of field isolations of the virus and/or susceptibility to infection and transmission rates following competence studies. Among the important *Culex* spp. recorded, *Culex zombaensis*, *Culex theileri* and *Culex pipiens* were included, which are very efficient vectors and known to play important roles during epizootics [26–31]. Also within the category of flood water *Aedes* mosquitoes known to contain the primary RVF vectors, *Aedes dentatus* was dominant with only low occurrence of *Aedes mcintoshi* and *Ae. tarsalis* which have been associated with isolation of the virus in Kenya and elsewhere [3, 5, 32]. Moreover, *Ae. dentatus* has been described as a potential epizootic and possibly reservoir vector of the virus [30].

Our results show that overall, attraction and engorgement rates were at least three fold higher for cow than goat or sheep. Mosquitoes find their hosts mainly through orientation to olfactory stimuli emanating from the host [33]. Our data suggest that the relatively larger size of the cow compared to the small ruminants sheep and goat could account for the cow releasing larger emissions of CO_2_, which act as a long distance attractant for host seeking mosquitoes [11, 34]. However, we found that the engorgement rate was clearly independent of the number attracted for certain mosquito species. This suggests that other factors such as host specific odours may contribute to the overall attraction of the host as has been shown in related studies [11, 34]. This pattern was very evident for most of the flood water *Aedes* spp., which contain most of the known primary RVF virus vectors. Irrespective of the catch size, the number that engorged was quite high up to 83 % and did not vary significantly across the hosts examined. This was the case for the species *Ae. dentatus* with a similar pattern observed even for species which were less abundant, such as *Ae. tarsalis* and *Ae. mcintoshi* (Table 4). Previous observations based on blood meal analysis have documented preferential feeding of flood water species of  *Aedes* (*Ae. mcintoshi*, *Ae. dentatus*, *Ae. cumminsi* and *Ae. sudanensis*) on cattle [6, 35, 36]. However, a related recent study has shown that some of these flood water *Aedes* spp. (*Ae. mcintoshi* and *Aedes ochraceus*) obtained bloodmeals in equal proportions from these vertebrate hosts with goat (*Capra hircus*) and cattle (*Bos taurus*) being the most common sources [7]. The finding that attraction and feeding success among these hosts did not vary suggest that feeding preference for these flood water *Aedes* spp. could largely be attributed to the composition and abundance/availability of these hosts and possibly other mammalian hosts in a given locality. This behaviour could potentially contribute to reproduction and effective survival of these mosquito species regardless of the local host population.

Most *Culex* spp. were consistently attracted to cow than to either sheep or goat. While attraction of *Culex* spp. on cow was highest, overall, only 45.6 % (592/1297) of the *Culex* spp. were engorged. Among the *Culex* spp. fairly captured across the different hosts, results showed that overall engorged rate varied from as low as 22.2 % for *Cx. univittatus* to 31.7 %, 37.0 %, 56.2 % and 68.7 % for *Cx. vansomereni*, *Cx. pipiens*, *Cx. zombaensis* and *Cx. terzii*, respectively. This clearly indicates that attraction does not always translate into feeding success. Although a number of *Culex* spp. prefer birds [37], it appears that these species can readily blood feed on a range of mammalian hosts. This could justify the role of some of these species as secondary vectors of RVFV acting as bridge vectors to extend infection even to humans given the commonality in host attractive cues among these mammals [15, 38].

The increasing association of *Anopheles* mosquitoes with arboviruses is of concern. RVFV and other arboviruses of medical importance such as Ngari virus, O'nyong-nyong, have been isolated from species such as *Anopheles gambiae* and *Anopheles funestus*, *Anopheles coustani*, *Anopheles squamosus* mosquitoes [3, 5, 39–41]. Engorged rates were highest overall among the anophelines (613/735) and dominated by *An. funestus* (86.9 %) followed by *An. gambiae* (78.3 %) and *An. coustani* (76.6 %). The finding confirms their high ability to feed on animals. This result may contrast earlier findings that have suggested high degrees of anthropophily [8, 42–44], a pattern that is likely biased by collections indoors or outdoors where humans and animals are dominant, respectively. Our findings, however, confirm their high ability to feed on animals especially on cows, although this may be associated with only certain species within the complexes that we did not delineate which is only possible *via* molecular means.

Our experimental design using animals allowed us to trap certain species in high numbers not readily collected using light traps. This was the case of the anophelines whose abundances are generally known to be underestimated using light traps [45, 46]. In fact, such decreased efficiency of light traps has been documented even in detecting the presence of anopheline species like *An. funestus* [46].

We did not evaluate the biting activity of the mosquitoes collected. Biting activity pattern during the day or night has been observed [18] although this varied depending on the species and the animal used as bait. In our mosquito counts, relatively few numbers of mosquitoes were collected at the sampling times (22:00 h) compared to 05:00 h (data not shown or captured). The engorged mosquitoes aspirated are more likely to have fed on that host as nearly 100 % of the female mosquitoes collected in the traps were freshly blood fed and in accordance with previous reports [47]. Also, delineating the member species of the *An. gambiae* complex and *An. funestus* group might be helpful in future studies to further ascertain the exact species-host feeding associations and potential involvement in disease transmission.

## Conclusion

In conclusion, an increase in the biting rate would be expected to result in increased pathogen transmission to susceptible hosts, all other conditions being equal. In the case of RVF amplifiers examined, we observed an overall increased attraction and engorgement of mosquitoes on cow relative to sheep and goat. This confirms higher biting pressure of the community of mosquitoes examined on cow. However, attraction did not always translate into feeding success and this latter most important epidemiological parameter did not seem to vary for specific species among the hosts notably the flood water *Aedes* spp., the primary vectors of RVFV. The overall high attractiveness of the mosquitoes to cow suggest when used as bait it can be exploited in the monitoring and control of disease-causing mosquitoes by incorporating say a tent impregnated with insecticide as in our experimental design. This approach can be employed as a push-pull intervention tool during arbovirus disease outbreaks to divert significant bites away from humans to livestock where they are then killed. Based on high attraction and engorgement to anophelines and particularly the malaria vectors, use of cows as bait may be a promising approach in the fight against malaria. This may be of great value against outdoor biting fractions which remains an important focus of sustaining malaria and out of reach of current indoor vector control tools such indoor residual spray (IRS) and long lasting insecticide treated bed nets.
